# Sepsis-induced changes in differentiation, maintenance, and function of memory CD8 T cell subsets

**DOI:** 10.3389/fimmu.2023.1130009

**Published:** 2023-01-23

**Authors:** Mohammad Heidarian, Thomas S. Griffith, Vladimir P. Badovinac

**Affiliations:** ^1^ Department of Pathology, University of Iowa, Iowa, IA, United States; ^2^ Department of Urology, University of Minnesota, Minneapolis, MN, United States; ^3^ Minneapolis Veterans Affairs Health Care System, Minneapolis, MN, United States; ^4^ Interdisciplinary Graduate Program in Immunology, University of Iowa, Iowa, IA, United States

**Keywords:** sepsis, memory, CD8 T cell, composition, differentiation, function, Immunoparalysis

## Abstract

Formation of long-lasting memory lymphocytes is one of the foundational characteristics of adaptive immunity and the basis of many vaccination strategies. Following the rapid expansion and contraction of effector CD8 T cells, the surviving antigen (Ag)-specific cells give rise to the memory CD8 T cells that persist for a long time and are phenotypically and functionally distinct from their naïve counterparts. Significant heterogeneity exists within the memory CD8 T cell pool, as different subsets display distinct tissue localization preferences, cytotoxic ability, and proliferative capacity, but all memory CD8 T cells are equipped to mount an enhanced immune response upon Ag re-encounter. Memory CD8 T cells demonstrate numerical stability under homeostatic conditions, but sepsis causes a significant decline in the number of memory CD8 T cells and diminishes their Ag-dependent and -independent functions. Sepsis also rewires the transcriptional profile of memory CD8 T cells, which profoundly impacts memory CD8 T cell differentiation and, ultimately, the protective capacity of memory CD8 T cells upon subsequent stimulation. This review delves into different aspects of memory CD8 T cell subsets as well as the immediate and long-term impact of sepsis on memory CD8 T cell biology.

## Introduction

Populations of memory CD8 T cells can be maintained for their entire lifetime of the host once formed, and these cells confer protection against intracellular infections and mediate antitumor immunity (1–5). Generation of these cells is an important objective for many vaccination strategies (6–9). Compared to their naïve counterparts, memory CD8 T cells typically exist at a much higher frequency, are localized to different lymphoid and non-lymphoid tissues throughout the body and have a less stringent activation mechanism (10–13). These characteristics allow memory CD8 T cells to quantitively and qualitatively mount a more robust immune response than naïve CD8 T cells, collectively resulting in more effective control of intracellular pathogens (14–16). Significant heterogeneity exists within the memory CD8 T cell pool at epigenetic, transcriptional, and protein expression levels prompting further classification based on their phenotype, localization, and function (17–21). Thanks to their durability and diverse subsets, memory CD8 T cells provide protective responses against reinfections even years after the initial challenge; however, the quantitative and qualitative changes experienced by memory CD8 T cells responses after the onset of a lymphopenic event such as sepsis remain to be fully understood.

Sepsis is defined as an exaggerated immune response to a systemic infection that leads to organ dysfunction (22). The disseminated infection initially triggers the exacerbated generation of an array of pro- and anti-inflammatory cytokines, collectively regarded as “cytokine storm” (23, 24). Most sepsis patients can now survive the acute phase of sepsis as recent advancements in critical care have alleviated the tissue/organ damage inflicted by the cytokine storm (25). However, transient lymphopenia and long-lasting immune dysfunction (termed ‘immunoparalysis’) follows the cytokine storm, rendering surviving patients more susceptible to secondary infections, viral reactivation, and decreased 5-year survival compared to non-septic patients (26–29).

Sepsis is a challenging health crisis affecting nearly 50 million people annually, with a mortality rate of approximately 20%. It disproportionally affects the elderly; 75% of sepsis-related mortality occurs in individuals above 65 (30–32). On the other hand, as individuals age, they accumulate more memory T cells due to vaccinations and (re)infections which is associated with decreased susceptibility to infections. In fact, memory CD8 T cells constitute more than two-thirds of the CD8 T cell population in adult humans (33–35). Tissue-wide presence of memory T cells and their crucial role in protecting against pathogens call for a detailed analysis of the impact of sepsis on memory T cells. Hence, investigating the short- and long-term effects of sepsis on memory T cells is imperative. In this review, we will first provide an overview of different subsets of memory CD8 T cells and how time and multiple antigen encounters influence their characteristics. We will then discuss the acute and sustained impairments of sepsis on memory CD8 T cells.

## Origin of memory CD8 T cells

Different models have been proposed to explain the origin and formation of antigen (Ag)-specific memory CD8 T (T_MEM_) cells following the rapid expansion/contraction of effector CD8 T cells (36, 37). One model argues for the linear differentiation of naïve CD8 T cells to effector CD8 T cells and then to memory CD8 T cells (38–43). An alternative model proposes memory CD8 T cells are directly derived from naïve CD8 T cells without undergoing the effector phase differentiation (44–46). Elegant human and murine studies have provided compelling evidence to support both models; however, one common theme between the two theories is that there exist two subsets of memory precursor (MP) or terminal effector (TE) CD8 T cells by which the former population gives rise to the memory pool and the latter is programmed to contraction (40, 41, 47). Presence and appropriate number of both subsets at the right time is crucial to clear the pathogen without causing immunopathology and generating a diverse memory pool for recall responses. MP and TE cells have been conventionally parsed out based on CD127 and KLRG1 expression. MP cells are CD127^hi^ and KLRG1^lo^, whereas TE cells are CD127^lo^ and KLRG1^hi^ (40), although recent work suggests a fraction of KLRG1^+^ effector cells can contribute to the memory pool (48–50). Nevertheless, the combination of Ag stimulation strength, inflammatory milieu, and tissue microenvironment alters Ag-specific CD8 T cell transcriptional programs, so that either subset is formed shortly after Ag encounter (15, 51–58). MP CD8 T cells express high levels of EOMES (59), FOXO1 (60), BCL-6 (61), ID3 (62), and TCF-1 (63, 64), whereas TE CD8 T cells express high levels of T-bet (40, 65), BLIMP-1 (66), ID2 (62), and Zeb2 (67). Each of these transcription factors (TF) plays a vital role in the formation, differentiation, and fate of effector cells. For example, Ag-specific CD8 T cells lacking EOMES or TCF-1 display diminished ability in differentiating to long-lasting memory CD8 T cells. In contrast, T-bet deficient CD8 T cells do not give rise to TE CD8 T cells (59, 63).

## Subsets of CD8 memory T cells

The first category of T_MEM_ cells (**Table 1**) is circulating memory (T_CIRCM_) CD8 T cells, which have been classically subdivided into two subsets of CD62L^lo^ CCR7^lo^ effector (T_EM_) and CD62L^hi^ CCR7^hi^ central memory (T_CM_) CD8 T cells (**Table 1** **)** (68). T_CIRCM_ CD8 cells can circulate between blood, secondary lymphoid organs, and non-lymphoid organs. However, the expression of lymph node homing receptors CCR7 and CD62L enhance the localization of T_CM_ cells in lymph nodes (LN) and white pulp of spleen, whereas T_EM_ cells are more prevalent in blood, red pulp of spleen, and non-lymphoid tissues (10, 68, 69). Functional studies have indicated both subsets are robust producers of IFN-γ and TNF-α in response to cognate Ag stimulation, but CD62L^+^ T_CM_ cells have enhanced proliferative potential and IL-2 production. In contrast, T_EM_ cells exhibit more efficient cytotoxicity and effector-like functions. The differential localization and functional abilities of T_CM_ and T_EM_ cells render each subset more effective against different pathogens, determined by the nature of infection elicited by each pathogen. For example, T_CM_ cells are more protective against LCMV-clone 13 and malignancies, while T_EM_ cells clear intracellular bacterium *Listeria monocytogenes* (LM) infections more efficiently (21, 70–73). Nevertheless, the distinct localization and functional abilities of T_EM_ and T_CM_ cells confer protection against a wide range of pathogens.

**Table 1:** Subsets of memory CD8 T cell pool and their characteristics.

Subset	Phenotype	Location	Function	Transcription Factors (TFs)
**T_CM_**	CD62L^hi^, CCR7^hi^, CD127^hi^ CD27^hi^, CX3CR1^lo^, KLRG1^lo^	Circulation, Primarily in LN and SLO	++ Ag-dependent expansion +/- Cytotoxicity	Eomes, FOXO1, Bcl6, Id3, TCF1
**T_EM_**	CD62L^lo^, CCR7^lo^, CD127^hi/lo^ CD27^hi/lo^, CX3CR1^hi/lo^ KLRG1^hi/lo^	Circulation, primarily in blood and occasionally NLT	+/- Ag-dependent expansion ++ Cytotoxicity	T-bet, Blimp1, Zeb2, Id2
**T_RM_**	CD69^hi^ depending on NLT: CD103^hi^ CD49a^hi^, CXCR3^hi^, CXCR6^hi^	Primarily NLTs, also found in draining LN	+ Proliferation ++ Sense and alarm function	Hobit, Blimp1, Runx3

+/- means a great fraction of the cells in the subset is endowed with the function while a noticeable population within the subset is not.

In addition to T_CIRCM_, tissue-resident memory (T_RM_) CD8 T cells are non-lymphoid tissue-restricted T_MEM_ cells that patrol tissues for pathogen invasion (**Table 1** **)** (74–76). These cells are typically situated in barrier sites and act as first responders upon Ag re-encounter with their sensing and alarm function; they mediate protection through cytotoxicity and/or secreting cytokines to recruit other immune cells to the site of pathogen invasion (75, 77–80). Although Hobit^+^ MP cells in non-lymphoid tissues (NLTs) are thought to be the major population contributing to the T_RM_ pool (76, 81, 82), it is not yet clear whether the potentiation of the effector cells to T_RM_ fate is induced either in the circulation prior to NLT recruitment or once located into NLT (83). T_RM_ cell fate requires downregulation of T-bet, EOMES, and TCF-1 to enable responsiveness to TGF-β, which signals for expression CD103, a critical tissue retention factor important in the generation of T_RM_ in epithelial tissue (58, 84, 85). Additionally, HOBIT/Blimp1 and Runx3 play a critical role in T_RM_ formation and differentiation (82, 86–88). ‘IV exclusion’ (89) and expression of tissue residence markers such as CD69 and CD103 are the most widely-used markers to distinguish T_RM_ cells from other T_MEM_ cells (76, 90). However, technically-challenging parabiosis experiments remain the gold-standard method to determine tissue residency (74, 91). Due to their strategic localization, which allows for early defense against pathogens, many studies have explored vaccination strategies that generate long-lasting T_RM_ cells to improve the efficacy of immunizations (92–98).

## Heterogeneity of T_CIRCM_ and T_RM_ cells

With the advent of multi-spectral flow cytometry and single-cell transcriptomics, the heterogeneity of both T_EM_ and T_CM_ populations has become more evident. CD62L^-^ T_CIRCM_ can further be subdivided into two populations of CD127^-^ CD27^-^ or CD127^+^ CD27^+^ subsets. The former subset is a descendant of KLRG1^+^ TE cells and termed long-lived effector cells (LLEC) (49) and/or terminally-differentiated effector memory cells (t-T_EM_) (50), as they express TE signature genes such as KLRG1 and CX3CR1 as well as some memory-signature genes such as Bcl2 and TCF-1. Compared to T_CM_ and CD127^+^ T_EM_ cells, t-T_EM_ cells demonstrate the highest expression of granzymes and provide robust protection in LM rechallenge models on a per-cell basis indicating superior cytolytic function, but t-T_EM_ cells show impaired IL-2 production and poor tumor control. Interestingly, once t-T_EM_ cells are parsed out of CD62L^-^ T_CIRCM_ and T_EM_ cells are redefined as CD127^+^ CD62L^-^ memory CD8 T cells, the functional differences between the redefined T_EM_ and CD62L^+^ T_CM_ cells are minimized. This suggests the t-TE_M_ cells that make up a significant population of CD62L^-^ T_CIRCM_ cells may drive the differences that have previously been reported with respect to proliferative and cytotoxic abilities of CD62L^+^ and CD62L^-^ T_CIRCM_ cells.

Recent studies have shed light on the heterogeneity within the T_CM_ population. A small subset of CD62L^+^ TCF1^+^ MP cells with restrained effector-phase proliferation and expression of inhibitory receptors have been identified to give rise to a multipotent subset of T_CM_ cells with superior recall responses (99), matching another finding where CD62L^+^ TCF1^hi^ MP cells form T_CM_ cells with stemness features (100). Additionally, a study by Bresser et al. suggests the replicative history of the T_CM_ pool dictates the transcriptional program and functionality of T_CM_ cells (101). Specifically, T_CM_ that have undergone fewer prior cell divisions demonstrate quiescence and stemness features with more efficient recall responses than the T_CM_ with more cell divisions which exhibit effector-like characteristics. The quiescent cells within the T_CM_ pool share features of self-renewal and multipotency with stem cell-like memory cells (T_SCM_) that remain poorly defined in murine models (42, 45).

Much of the heterogeneity described to the T_RM_ population is attributed to the distinct tissue microenvironment that T_RM_ cells are exposed to from tissue to tissue (102–104). Differential microenvironmental features lead to the phenotypic and transcriptomic alterations during the generation, differentiation, and maintenance of T_RM_ cells found in different organs, even in the same infectious model (105). This is well-reflected in the distinct T_RM_ markers and tissue-specific retention proteins; for example, despite the uniform expression of CD69 by T_RM_ cells in different tissues, expression of CD103, adhesion molecule CD49a, and chemokine receptors CXCR3 and CXCR6 are variable (103, 104). Notably, the heterogeneity of T_RM_ cells from different tissues is not limited to surface markers. It is also observed in transcriptional makeup and genome accessibility as tissue milieu instructs T_RM_ cells with a transcriptional network required for specific tissue adaptation (76, 105). Recent work also suggests T_RM_ cells within the small intestine could be further subdivided into stem-like Id3^hi^ T_RM_ and effector-like Id3^lo^ T_RM_ cells with differential multipotency and effector function capacity (106). Nevertheless, more studies are needed to fully delineate the heterogeneity within T_RM_ pool.

## Evolution of the T_MEM_ pool after multiple antigen encounters

One hallmark of T_MEM_ cells generated *via* infection and/or vaccination is their ability to maintain their number and function for the life of the individual. The durability of T_MEM_ in an Ag-independent fashion relies on homeostatic signals from IL-7 and IL-15 that promote memory T cell survival (107). Despite their relative numerical stability, the CD8 memory pool undergoes significant transcriptional and phenotypic changes over time. With increasing time, the frequency of T_CIRCM_ cells expressing TCF1, Bcl6, Id3, and EOMES and long-term memory maintenance genes such as CD27, CD127, and CD122 increases while the expression of T-bet, Zeb2, Runx1, and Id2 and effector-like genes such as CX3CR1 and KLRG1 decreases. At an early memory timepoint, T_EM_ cells with high expression of effector-like genes are the dominant subset of T_CIRCM_; however, superior hemostatic proliferative capacity of T_CM_ cells and/or direct conversion of CD127^+^ CD62L^-^ T_EM_ cells to T_CM_ cells results in gradual increase in T_CM_ representation over time. This results in late T_CIRCM_ cells to possess greater capacity for IL-2 production, secondary expansion, and higher order memory potential than early T_CIRCM_ cells (5, 21, 36, 108). On the other hand, T_RM_ cells of distinct tissues exhibit differential longevity; lung T_RM_ cells wane over time resulting in loss of protection (109, 110) while skin T_RM_ cells persist for a long time with robust protective function (111). Nevertheless, few studies have examined the impact of time on the phenotype and function of T_RM_ cells.

Following pathogen re-encounter and secondary expansion of primary (1°) T_CIRCM_ cells, secondary (2°) T_CIRCM_ cells are generated which can give rise to higher order T_CIRCM_ cells upon additional Ag encounter. Higher order T_CIRCM_ cells display differential tissue localization, phenotypic, and functional characteristics than 1° T_CIRCM_ cells. With increasing number of Ag stimulations, higher order T_MEM_ cells become more cytolytic with greater ability in trafficking to peripheral tissues, but reduced progression to a T_CM_ phenotype, responsiveness to homeostatic cues, and proliferative capacity (112–116). ‘T_EM_ -like’ features of higher order T_MEM_ cells render this population more protective than 1° T_MEM_ cells against pathogens, such as LM, that primarily infect and localize to peripheral tissues (73, 117). Although the more Ag encounters T_MEM_ cells experience, the more they become phenotypically and functionally like T_EM_ cells, gene set enrichment analysis (GSEA) shows no progressive enrichment in T_EM_-associated genes in 2°, 3°, or 4° T_MEM_ cells (118). Hence, repeated Ag stimulation induces major changes in gene expression patterns of individual cells as opposed to merely changing the T_EM_ : T_CM_ ratio.

Antigenic challenge induces robust cytokine response from 1° T_RM_ cells which recruits immune cells including T_RM_ precursors to the site of infection to generate more T_RM_ population. Data suggested that 1° T_RM_ cells could also proliferate upon reinfection to give rise to 2° T_RM_ cells (119, 120); however, recent findings provided evidence that different subsets of T_RM_ possess different proliferation capacity. Using a fate-mapping system to track CD103-expressing CD8 T cells, von Hoesslin et al. and Fung et al. showed that CD103^+^ T_RM_ cells have limited proliferation capacity, but CD103^-^ T_RM_ cells undergo robust expansion upon Ag re-encounter, further highlighting the heterogeneity within T_RM_ pool (121, 122). Nonetheless, successive Ag exposures improve the longevity and protective function of T_RM_ pool; for example, 4° influenza-specific T_RM_ cells show enhanced durability and heterosubtypic immunity than 1° T_RM_ cells (123). This is attributed to continuous localization of 4° T_EM_ cells to lungs followed by subsequent conversion to T_RM_ cells. Several studies have reported lymph node T_RM_ cells in the context of skin and lung infections (124, 125) and Ag re-encounter may lead to migration of T_RM_ offspring to the draining lymph node (125). Similarly, repeated Ag exposures result in higher lymph node T_RM_ cells and increased representation of CD103^+^ CD69^+^ LN T_RM_ cells, leading to better local protection than 1° T_RM_ cells (126). Overall, repetitive Ag encounter consolidates the T_RM_ memory pool through the formation of higher order T_RM_ cells and/or differentiating pre-existing T_CIRCM_ to T_RM_ cells upon recruiting to the tissue.

## Short- and long-term impact of sepsis on the composition of T_MEM_ pool

**Figure 1:**
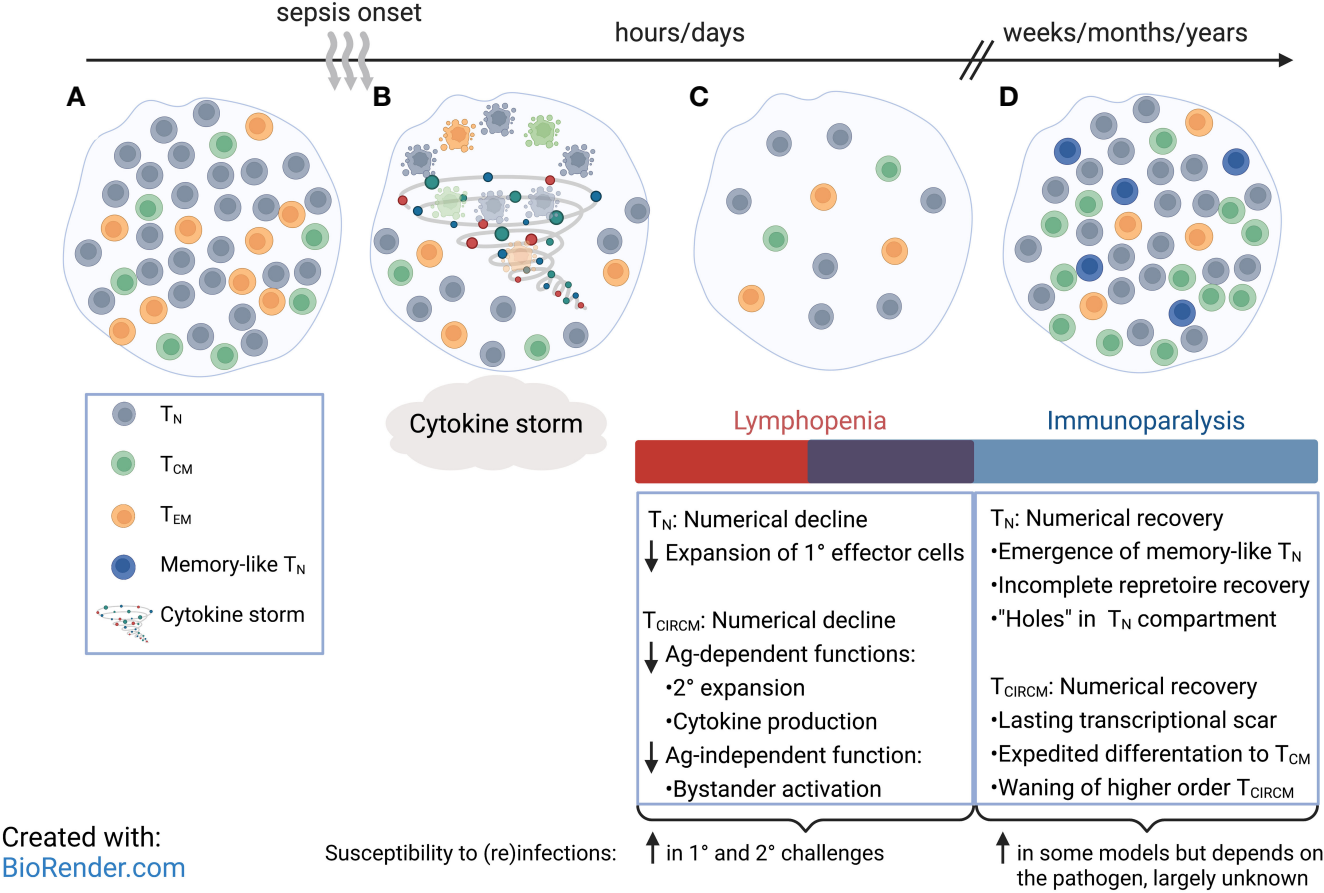
Compositional and phenotypical changes of circulatory CD8 T cell pool after sepsis. **(A)** Circulatory CD8 T cell pool consists of naïve CD8 T (T_N_) cells and memory CD8 T (T_CIRCM_) cell subsets. **(B)** Increased levels of circulating pro- and anti-inflammatory cytokines mark the initial phase of a septic insult, followed by induction of apoptosis in CD8 T_N_ and T_CIRCM_ in a stochastic manner. **(C)** Rapid loss of CD8 T_N_ and T_CIRCM_ and other lymphocytes result in transient lymphopenia, accompanied with early signs of immunoparalysis. **(D)** Number of CD8 T_N_ and T_CIRCM_ return to pre-sepsis values; however, some CD8 T_N_ express memory-like phenotype, and the central memory CD8 T (T_CM_) cells are enriched over effector memory CD8 T (T_EM_) cells. Many patients continue to suffer from a long-lasting state of immunoparalysis.

Sepsis significantly reduces the number of lymphocytes (127–130), including CD8 T cells, *via* apoptosis (131–133). While naive (T_N_) CD8 T cells are more susceptible to radiation-induced apoptosis and are lost to a greater extent than T_CIRCM_ cells (134), both T_N_ and T_CIRCM_ cells display similar susceptibility to the sepsis-induced numerical decline (135–137). Additionally, further investigation into the subset composition of T_CIRCM_ cells before and after sepsis reveals the numerical decline of T_CM_ is equal to that of CD62L^-^ T_EM_ cells. Hence, sepsis stochastically targets CD8 T cells, and all circulating CD8 T cells are lost in a non-discriminatory fashion regardless of their antigen exposure history (**Figures 1B, C** **)** (135, 137). Indeed, this interpretation is validated as 1° and 4° T_CIRCM_ cells exhibit similar fold loss following a septic event (138).

**Figure 2:**
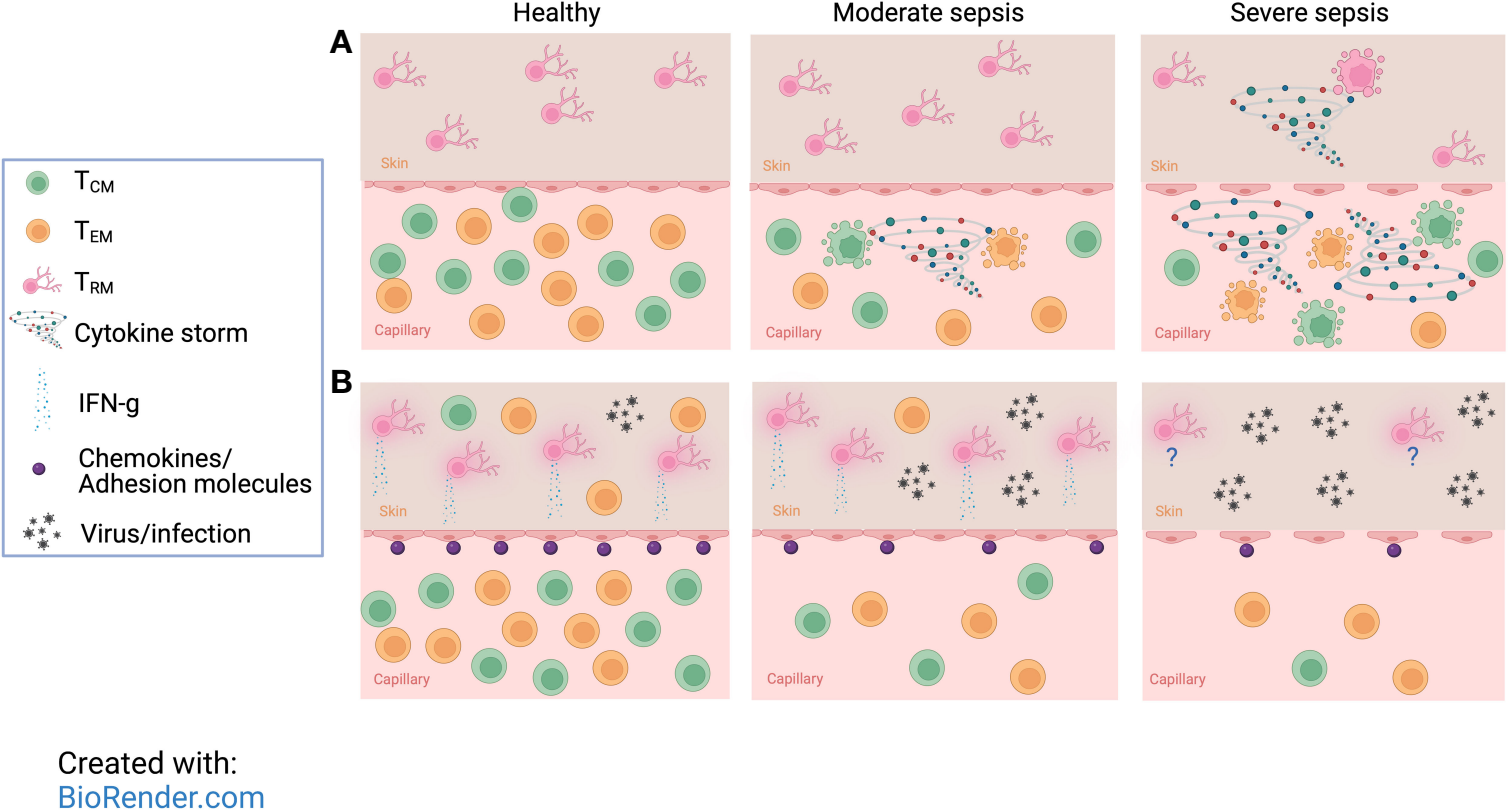
Severe sepsis imposes more drastic numerical and functional diminishment in memory CD8 T cells than moderate sepsis. **(A)** Despite rapid loss of CD8 T_CIRCM_, undamaged endothelial barriers protect tissue-resident memory CD8 T (T_RM_) cells from moderate sepsis-induced apoptosis. However, severe sepsis not only causes a more drastic decline in number of T_CIRCM_, but it also overcomes the endothelial barrier and T_RM_ become vulnerable to detrimental effects inflicted by the sepsis-induced cytokine storm resulting in rapid apoptosis of T_RM_ cells. **(B)** Moderate sepsis does not change the number and per cell function of T_RM_ cells, but it reduces the ability of endothelial cells to upregulate chemokines and adhesion molecules in response to T_RM_-derived cues which leads to reduced recruitment of effector cells and poor protection against localized rechallenges. With increasing severity of sepsis, the protection against localized reinfections is even more compromised due to reduced number of T_CIRCM_ and T_RM_. This figure was designed using “The Inflammatory response” template available at BioRender.com.

Unlike T_CIRCM_ cells, T_RM_ cell numbers remain unchanged following sepsis-induction that leads to low mortality levels (0-20% - moderate sepsis). Using a vaccinia infection model to generate T_CIRCM_ and T_RM_ with the same Ag specificity, we found the number of ‘IV positive’ T_CIRCM_ cells significantly declined after moderate sepsis, but the number of ‘IV negative’ skin T_RM_ cells were held constant (**Figure 2A**, middle) (137, 139). Interestingly, T_RM_ cells within tumors and non-lymphoid organs are also more protected from radiation-induced cell death than circulatory T cells (140). Two explanations were postulated to justify the resistance of T_RM_ cells to sepsis-induced apoptosis. One is that T_RM_-specific factors may protect this subset from sepsis-mediated apoptosis, as T_RM_ and T_CIRCM_ cells are phenotypically and transcriptionally distinct. Alternatively, the local environment in which T_CIRCM_ and T_RM_ cells reside may predispose one subset to sepsis-induced apoptosis but protect the other. Specifically, T_RM_ cells that reside in NLTs and have limited access to circulation may be more protected from the cytokine storm than the T_CIRCM_ cell typically found in blood and SLO. While the first explanation has yet to be examined, the second one was tested elegantly through varying the severity of sepsis. To do so, the cecal ligation and puncture (CLP) method with one or two punctures was implemented to recapitulate moderate or severe sepsis, respectively (141, 142). Moderate CLP-induced sepsis did not inflict enough damage to increase endothelial vascular permeability and leakage of cytokine storm to NLTs; however, severe sepsis led to a disruption of the endothelial barrier exposing the once-shielded NLT to pro- and anti-inflammatory cytokines (and other proteins and metabolites). Therefore, severe sepsis not only instigates a more dramatic T_CIRCM_ cell loss compared to moderate sepsis, but it also results in a significant decline in the number of T_RM_ cells (**Figure 1A**, right) (142). Overall, these data demonstrate T_CIRCM_ and T_RM_ cells display differential susceptibility to sepsis due to their distinct anatomical localization.

Sepsis-induced lymphopenia is a transient event, and lymphocyte numbers will eventually return to pre-sepsis levels. However, there is limited information detailing the mechanisms responsible for the numerical restoration and the long-term impact of sepsis on T cell biology. Longitudinal studies using TCR-transgenic CD8 T cells (i.e., P14) adoptively transferred into C57/Bl6 recipients have shown that the number of both T_N_ and T_CIRCM_ cells quickly bounce back to the pre-sepsis baseline state. Lymphopenia-induced proliferation is thought to drive the numerical recovery of T_N_ and T_CIRCM_ cells as IL-7 and IL-15 mediate rapid proliferation of surviving lymphocytes to fill the empty space. Increased frequency of Ki-67^+^, marker for cell cycling and a non-G_0_ status, T_N_ and T_CIRCM_ cells in both murine and human septic samples provides evidence for increased proliferation of CD8 T cells after resolution of the acute phase of sepsis (143, 144). Recent murine and clinical studies have exploited the pro-survival features of IL-7 on T cells, as IL-7 treatment alleviates sepsis-indued T cell loss *via* preventing apoptosis and accelerating numerical recovery of lymphocytes (145–147). This notion has opened new lines of investigation to explore the therapeutic effects of IL-7 and other cytokine complex treatments in ameliorating sepsis-induced immune dysfunction.

Despite apparent numerical recovery of T_N_ cells, the composition and phenotype of the post-sepsis T_N_ pool is altered. Reduced primary effector responses in the post-septic host indicates an incomplete repertoire recovery and a less diverse T_N_ pool. This is indeed the case for naïve Ag-specific CD4 T cells (148), but it remains to be determined if the same thing occurs for CD8 T cells. In addition, some studies suggest post-sepsis T_N_ cells have increased expression of memory-associated markers, such as CD11a, for an unknown period (**Figure 1D** **)** (143). These observations have prompted more detailed investigation into long-term impact of sepsis on the numerically recovered T_MEM_ compartment.

Transcriptional analysis of T_CIRCM_ cells from sepsis survivors indicates that sepsis causes a long-lasting ‘transcriptional scar’ in T_CIRCM_ cells by inducing transcriptional changes both immediately after onset of sepsis and during the recovery phase. Specifically, T_CIRCM_ cells from CLP hosts show upregulation of pathways that work in concert to aid in cell cycling and increase the proliferation output long after sepsis induction. Additionally, T_CIRCM_ transcripts from sham hosts are more effector-like whereas T_CIRCM_ transcripts from CLP hosts are enriched in sets of genes associated with long-term memory, pointing to potential composition differences between the two groups. Indeed, the post-sepsis environment greatly shapes the phenotype and the composition of T_CIRCM_ pool. Precisely, the numerical recovery of T_CIRCM_ cells is accompanied with increased representation of T_CM_ cells, the memory subset with highest proliferation capacity (**Figure 1D** **)**. Examining the effector and memory-related markers shows the enrichment of CD62L^+^ KLRG1^-^ CD127^+^ CX3CR1^-^ T_CIRCM_ cells in the septic host. The enrichment of T_CM_ cells is ascribed to the enhanced capacity of T_CM_ cells to sense lymphopenia-induced homeostatic cues that trigger rapid cell cycling and enrichment of T_CM_ cells in the T_CIRCM_ pool (144). Taken together, despite equal susceptibility of T_CIRCM_ subsets to sepsis, surviving T_CIRCM_ cells with greater homeostatic proliferation potential preferentially repopulate the lymphopenic space leading to long-lasting altered T_CIRCM_ subset composition.

1° T_CIRCM_ cells are not the only T_MEM_ cells affected by sepsis. Our lab has recently demonstrated that higher order T_CIRCM_ cells are equally susceptible to the sepsis-induced death as 1° T_CIRCM_ cells. This is particularly important as the human population, especially the elderly with the highest susceptibility to sepsis complications, is seeded with a diverse pool of T_MEM_ cells and different Ag exposure histories. Additionally, we speculated the diminished baseline proliferative capacity of higher order T_CIRCM_ cells vs. 1° T_CIRCM_ cells leads to preferential numerical recovery of 1° T_CIRCM_ cells and dilution of higher order T_CIRCM_ cells post-sepsis. Examining Ki-67 expression and BrdU incorporation of 1° and 4° T_CIRCM_ cells revealed that unlike in 1° T_CIRCM_ cells, sepsis did not invoke vigorous proliferation in 4° T_CIRCM_ cells. Subsequently, the frequency of 4° T_CIRCM_ cells progressively decreased while 1° T_CIRCM_ increased resulting in a less diverse T_CIRCM_ pool. Despite triggering rapid proliferation of 1° T_CIRCM_ cells, administration of IL-7 did not boost the numerical restoration of 4° T_CIRCM_ cells which further capitalizes the accumulation of 1° T_CIRCM_ cells after sepsis (138). Overall, the post-sepsis environment favors the repopulation of T_CIRCM_ cells with high proliferative capacity, leading to altered subset composition and reduced heterogeneity within the T_CIRCM_ pool.

## Short- and long-term impact of sepsis on the function of T_MEM_ pool

Increased susceptibility of sepsis survivors to previously-encountered pathogens and viral reactivation insinuates compromised protection conferred by T_MEM_. The impact of sepsis on the protective capacity of T_MEM_ can be dissected at different levels because the ‘per cell’ functional fitness (such as cytolytic capacity and cytokine secretion) of T_MEM_ cells is key in mediating pathogen clearance, in addition to their number, tissue localization, and ability to communicate with other cells being crucial for mounting a protective immune response. Thus, we will next discuss the immediate effect of sepsis on functional capacity of different subsets of the T_MEM_ pool and finish with a description of the long-term impact of sepsis on T_MEM_ -mediated immunity.

Lymphopenia is not the only immunological catastrophe that a septic host experiences shortly after the onset of sepsis. Sepsis impairs the Ag-dependent functions of T_CIRCM_ on a per cell basis. Particularly, sepsis diminishes the IFN-γ production in response to cognate Ag resulting in decreased Ag sensitivity and functional avidity of T_CIRCM_ cells. In response to the cognate antigen, the compromised cytokine production and proliferative capacity of T_CIRCM_ render septic hosts more susceptible to homologous reinfections. Nevertheless, T_MEM_ cells do not mediate protection only in presence of their cognate Ag. When T_MEM_ are ‘bathed’ in a highly inflammatory environment, they are activated to produce more cytokines and cytotoxic granules such as granzyme B. This ‘bystander activation’ of T_MEM_ is Ag-independent, but inflammation-dependent (149–152). Interestingly, sepsis also impairs the Ag-independent functions of T_MEM_. In response to a heterologous infection, upregulation of activation markers and granzyme B was compromised in T_CIRCM_ of CLP hosts (135). Together, these results suggest sepsis impairs the Ag-dependent and -independent functions of T_CIRCM_ through influencing T-cell intrinsic and extrinsic factors.

Due to their localization to NLTs and being shielded from the damages of moderate cytokine storm, T_RM_ maintain their numbers, and their ‘sensing and alarm’ function as measured by IFN-γ production in response to Ag stimulation (**Figure 2B**, middle). Surprisingly, despite the intact number and function of T_RM_ in the post-septic host, the protective capacity of T_RM_ is diminished after moderate sepsis. In vaccina virus (VacV)-immune mice that underwent either CLP or sham surgeries, CLP hosts showed sustained high viral load and inability to clear VacV after re-challenge (139). Interestingly, this finding is contrary to other data suggesting T_RM_ confer better protection than T_CIRCM_ against VacV reinfections (74). This difference raises the question as to how sepsis diminishes the protective capacity of T_RM_ despite their unchanged numbers and function. Subsequent investigation revealed that sepsis decreases the ability of vascular endothelium to express chemokines and adhesion molecules in response to T_RM_ inflammatory cues (**Figure 2B**, middle), resulting in the inefficient recruitment of effector cells to the site of pathogen invasion and ultimately poor pathogen control (139). In severe sepsis, the numerical decline of T_RM_ further exacerbates the diminished protection in localized reinfections (**Figure 2B**, right) (142). Collectively, these results suggest sepsis diminishes T_RM_ recall responses through disrupting their ability to recruit effector cells.

How tissue-specific factors contribute to the resistance of T_RM_ cells to moderate sepsis-induced cell death and functional impairment remains elusive. Blockade of TGF-β has been shown to render tumor T_RM_ cells more susceptible to radiation-induced numerical decline (140); hence, the potential role of TGF-β signaling in maintaining T_RM_ number and function after moderate sepsis should be explored. Additionally, the impact of sepsis on T_RM_ cells within NLTs other than skin and SLO T_RM_ cells in draining LN should be further investigated. While the data from our laboratory suggest that skin T_RM_ cells that are anatomically separated from circulation are numerically and functionally protected from moderate sepsis, the crosstalk of SLO T_RM_ cells with circulatory factors and the increased exposure of liver T_RM_ cells to blood may increase the sensitivity of SLO and liver T_RM_ cells to sepsis-mediated numerical loss and dysfunction. On the other hand, one could also argue for presence of shared T_RM_-specific factors that protect T_RM_ cells found in different tissues from moderate sepsis regardless of their localization.

Our discussion so far has focused on describing the functional impairments with the CD8 T cell compartment that ensue after septic insult. While they shed light on factors contributing to the increased susceptibility of septic hosts early after the insult, a noticeable percentage of sepsis survivors suffer from long-lasting immunoparalysis. Our studies on the T_CIRCM_ pool long after sepsis suggest the impairment in cytokine production after restimulation is resolved. In fact, a higher frequency of T_CIRCM_ from CLP hosts produce IL-2 in response to Ag stimulation when examined 30 days post-sepsis. Increased IL-2 production aligns with the enrichment of T_CM_ in the T_CIRCM_ pool at a late time post sepsis, as these cells have better IL-2 production than T_EM_. However, the preferential skewing of T_CIRCM_ pool by cells with the greatest proliferative capacity (i.e., 1° T_CM_) results in the reduced prevalence of T_CIRCM_ cells with greatest cytotoxic function (T_EM_ and higher order T_CIRCM_ cells) (138, 144). Enrichment of T_CM_ negatively impacted the ability of CLP hosts to clear pathogens in a LM rechallenge model (144). Additionally, recent studies have identified T_EM_ as the population seeding T_RM_ pools (109). T_CM_ overrepresentation may affect the maintenance of the T_RM_ pool by decreasing the supply of T_EM_. Overall, a memory pool with a diverse (but balanced) subset of cells is needed for the host to mount the most robust immune response possible. Enrichment of a subset of T_MEM_ at the expense of other subsets may substantially affect the overall fitness of T_MEM_ pool as each subset possesses a specialized role and function.

## Conclusion

Sepsis research has shifted focus to characterizing the factors leading to the long-lasting state of immunoparalysis that emerges following the resolution of acute phase of sepsis. Since sepsis survivors show increased susceptibility to secondary and recurring infections, these studies demand an in-depth analysis of the impact of sepsis on memory lymphocytes – the body’s most potent weapon in fighting against reinfections. Circulating memory CD8 T cells undergo substantial numerical attrition and functional impairment shortly after a septic insult deriving the host susceptible to heterologous and homologous reinfections. Additionally, tissue-resident memory CD8 T cells also display a diminished ability in recruiting effector cells in response to localized re-infections. Despite the apparent numerical recovery and per cell function, circulatory memory CD8 T cells demonstrate long-lasting changes in their transcriptional and epigenetic programs after sepsis resolution, with the most proliferative subset being overrepresented over time. Therefore, sepsis ultimately leads to altered subset composition and reduced heterogeneity in memory CD8 T cells in the circulation. Further investigation is required to delineate the long-term sepsis-induced changes in function and maintenance of tissue-resident memory CD8 T cells.

## Author contributions

All authors listed have made a substantial, direct, and intellectual contribution to the work and approved it for publication.
